# Supplementary material for: Splice-Junction-Based Mapping of Alternative Isoforms in the Human Proteome
Source: Cell Rep. Author manuscript; Available in PMC 2020 Jan 15. (PMC6961840; doi:10.1016/j.celrep.2019.11.026)
Supplement: 2 [file NIHMS1546469-supplement-2.zip › DF1/PXD009737/TestisTrypsin/TestisTrypsin_41_ARHGEF11_SSSQSIKPGNVR.pdf]

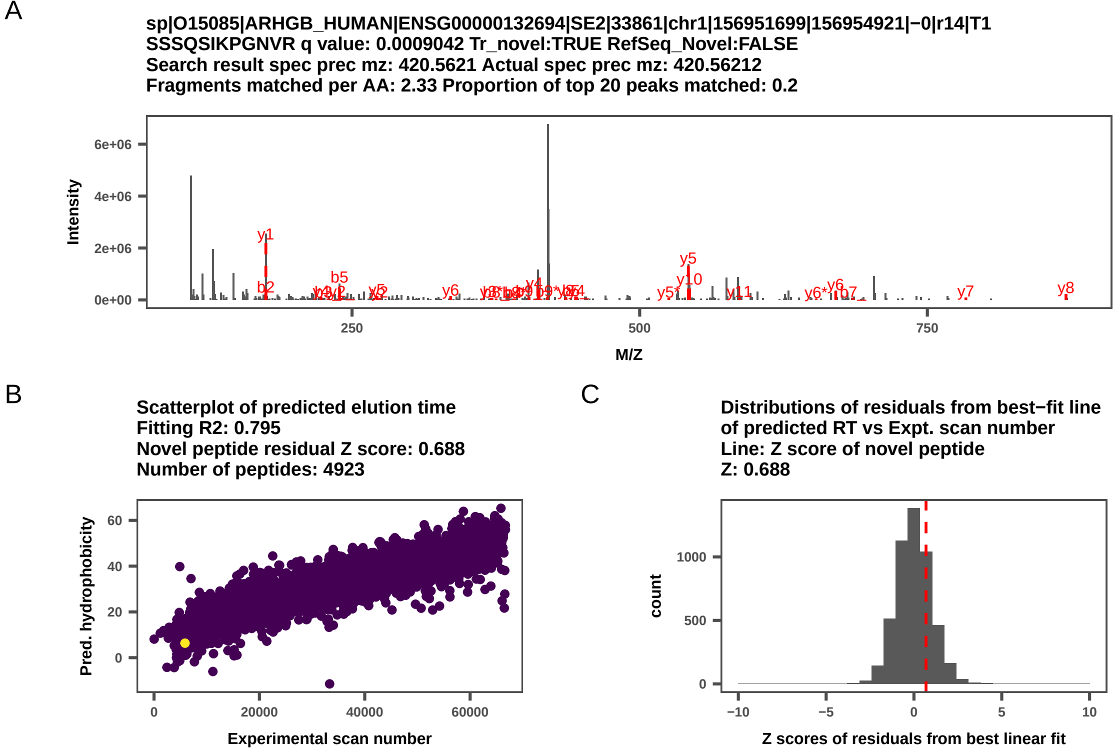
A
sp|O15085|ARHGB_HUMAN|ENSG00000132694|SE2|33861|chr1|156951699|156954921|−0|r14|T1
SSSQSIKPGNVR q value: 0.0009042 Tr_novel:TRUE RefSeq_Novel:FALSE
Search result spec prec mz: 420.5621 Actual spec prec mz: 420.56212
Fragments matched per AA: 2.33 Proportion of top 20 peaks matched: 0.2
Intensity
6e+06
4e+06
2e+06
0e+00
y1
b2
b5
y6
y5
y10
y5*
y11
y6*
y6
b7
y7
y8
250
500
750
M/Z
B
Scatterplot of predicted elution time
Fitting R2: 0.795
Novel peptide residual Z score: 0.688
Number of peptides: 4923
Pred. hydrophobicity
60
40
20
0
0
20000
40000
60000
Experimental scan number
C
Distributions of residuals from best−fit line
of predicted RT vs Expt. scan number
Line: Z score of novel peptide
Z: 0.688
count
1000
500
0
−10
−5
0
5
10
Z scores of residuals from best linear fit
